# Hibernation in a primate: does sleep occur?

**DOI:** 10.1098/rsos.160282

**Published:** 2016-08-10

**Authors:** Marina B. Blanco, Kathrin H. Dausmann, Sheena L. Faherty, Peter Klopfer, Andrew D. Krystal, Robert Schopler, Anne D. Yoder

**Affiliations:** 1 Duke Lemur Center, 3705 Erwin Road, Durham, NC 27705, USA; 2 Department of Functional Ecology, Hamburg University, Hamburg, Germany; 3 Department of Biology, Duke University, Durham, NC, USA; 4 Department of Psychiatry and Behavioral Sciences, Duke University School of Medicine, Durham, NC, USA

**Keywords:** REM, non-REM, metabolic rates, primate, lemur, hibernation

## Abstract

During hibernation, critical physiological processes are downregulated and thermogenically induced arousals are presumably needed periodically to fulfil those physiological demands. Among the processes incompatible with a hypome tabolic state is sleep. However, one hibernating primate, the dwarf lemur *Cheirogaleus medius*, experiences rapid eye movement (REM)-like states during hibernation, whenever passively reaching temperatures above 30°C, as occurs when it hibernates in poorly insulated tree hollows under tropical conditions. Here, we report electroencephalographic (EEG) recordings, temperature data and metabolic rates from two related species (*C. crossleyi* and *C. sibreei*), inhabiting high-altitude rainforests and hibernating underground, conditions that mirror, to some extent, those experienced by temperate hibernators. We compared the physiology of hibernation and spontaneous arousals in these animals to *C. medius*, as well as the much more distantly related non-primate hibernators, such as Arctic, golden-mantled and European ground squirrels. We observed a number of commonalities with non-primate temperate hibernators including: (i) monotonous ultra-low voltage EEG during torpor bouts in these relatively cold-weather hibernators, (ii) the absence of sleep during torpor bouts, (iii) the occurrence of spontaneous arousals out of torpor, during which sleep regularly occurred, (iv) relatively high early EEG non-REM during the arousal, and (v) a gradual transition to the torpid EEG state from non-REM sleep. Unlike *C. medius*, our study species did not display sleep-like states during torpor bouts, but instead exclusively exhibited them during arousals. During these short euthermic periods, non-REM as well as REM sleep-like stages were observed. Differences observed between these two species and their close relative, *C. medius*, for which data have been published, presumably reflect differences in hibernaculum temperature.

## 1. Introduction

When neither food nor water is sufficient, mammals must either migrate or severely depress their metabolic processes to survive. By expressing torpor, a number of species in a wide range of orders, from Monotremes to Primates, allow their core temperatures to be driven by the environment [1,2]. Thus, they eliminate the major component of metabolic demands: euthermia, or a continual maintenance of continuously elevated body temperature. When torpor persists over many days, we refer to it as hibernation.^1^ Whether this ability is a conserved or a convergent trait is still a matter of debate [4–7]. One recently emerged hypothesis proposes that the ability to reduce metabolism (and hide underground) is deeply rooted in mammalian phylogeny, and was critical for the survival of small mammals after the cataclysmic Cretaceous/Tertiary (K-T) extinction events—a period characterized by intense thermal radiation [8,9]. This would place the origins of hibernation in ‘tropical’ environments rather than the cold habitats we tend to associate hibernation with today.

Both temperate and tropical hibernators rely on hypometabolic states to save energy (and, in some instances to conserve water), as metabolic processes are temperature-dependent (but see the case of hibernating bears [10]). Simply stated, during torpor bouts, an individual's body temperature approximates that of the immediate environment (i.e. the cooler the hibernaculum, the lower the temperature, the lower the metabolism). Small-bodied Arctic hibernators may be an exception, as they must defend a minimum body temperature despite large gradients between ambient temperature (*T*_am_) and body temperature (*T*_b_) [11]. Most generally, whereas individuals exposed to cold temperatures during hibernation undergo thermogenically induced arousals to achieve euthermia and resume physiological processes slowed down by hibernation, on some occasions, tropical hibernators may achieve high body temperature by thermo-comforting inside poorly insulated hibernacula. Thus, hibernaculum temperature not only affects the frequency of arousals but it also determines whether arousals must occur at all [12–14].

Among those physiological demands restored during the arousal period, sleep appears to be critical. Early work with golden-mantled ground squirrels (*Callospermophilus lateralis*) showed that individuals artificially induced to hibernate at relatively warm temperature (e.g. 22°C) did sleep, supporting the claim that hibernation was an extension of slow-wave sleep [15]. In fact, these results favoured the idea that sleep was a pre-adaptation for hibernation [16]. However, these studies also showed that when brain temperature decreased to levels found in natural hibernation (approx. 6°C), brain electric activity became undetectable, which meant that sleep could not occur.

Additional work indicated that when core temperatures drop below 20°C, brain activity subsides to a level that no longer supports the neural discharges associated with sleep ([15,17], but see [18]). Prolonged sleep deprivation is followed by a loss of the ability to thermoregulate, severe cognitive impairments and, in some instances, death [19]. A linkage between sleep deprivation and euthermia was further supported by studies that evaluated the pattern of electroencephalographic (EEG) slow-wave activity (SWA) (measured as Delta Power) occurring after arousal from hibernation. Delta Power reflects the degree of homeostatic sleep drive resulting from sleep deprivation. During their spontaneous arousals, Arctic ground squirrels (*Urocitellus parryii*) and golden-mantled ground squirrels displayed a particular pattern of EEG SWA consistent with sleep deprivation. Importantly, the degree of SWA was proportional to the duration of the last hibernation bout (i.e. the extent of SWA clustering increased as a function of sleepless periods) [17,20]. European ground squirrels (*Spermophilus citellus*) hibernating at constant temperature also displayed increased EEG Delta Power in the early part of the night, which was proportional to the intensity of prior hibernation bouts [21].

However, other studies contradicted these findings. For instance, follow-up studies of European ground squirrels failed to show a delay or increase in EEG Delta Power when sleep was artificially prevented from occurring during the spontaneous arousals, as would be expected in a rebound recovery response due to sleep deprivation [22,23]. Moreover, golden-mantled ground squirrels displayed a negative correlation between SWA and brain temperature but not with duration of hibernation bouts [24]. As a result, it remains unclear if the spontaneous arousals are needed in order to satisfy a need for sleep which is not met during hibernation [25].

All of these studies have been carried out in non-primate temperate hibernators. Within primates, obligate hibernation has so far only been described in dwarf lemurs (genus *Cheirogaleus*), a group of small-bodied (130–600 g), highly frugivorous, night-active Malagasy lemurs. Despite living in tropical environments, these lemurs are confronted with similar challenges to those of their Arctic and temperate counterparts, such as resource scarcity and cold temperatures during the dry winter season. For instance, the fat-tailed dwarf lemur (*Cheirogaleus medius*), inhabiting dry forests, exclusively hibernates in tree hollows [12,26], whereas the ‘highland’ *Cheirogaleus* (e.g. *C, sibreei*) retreats underground during the hibernation season [13]. Thus, the animal's body temperature and metabolic rate can remain almost constantly low during hibernation (e.g. underground sites or hollows in large trees), or fluctuate according to the insulation properties of tree hollows. If dwarf lemurs can reach body temperatures above 30°C by passively tracking ambient temperatures, they avoid endogenously generated arousals; if dwarf lemurs hibernate underground in colder areas, they actively undergo energetically expensive arousals about once a week [12,26].

We have previously reported on studies of hibernation in *C. medius*, which we studied both in Madagascar, as well as at the Duke Lemur Center, NC, USA [27,28]. *Cheirogaleus medius* hibernates at temperatures between 10 and 30°C, for periods of up to seven months a year in the wild [12]. The EEG of hibernating *C. medius*, passively warmed without expressing endogenously generated arousals, is marked by rapid-eye movement (REM) sleep-like periods and an absence of non-REM sleep when body temperature is above approximately 25°C. Sleep does not occur at lower temperatures [28]. These findings were at odds with those from Arctic ground squirrels under laboratory conditions (23°C), where periods of such REM-like activity were absent during hibernation bouts [15].

Here, we provide data obtained during hibernation in two related species of dwarf lemurs, *C. crossleyi* and *C. sibreei*, which inhabit the colder uplands of Madagascar and hibernate underground, rather than in tree hollows [13]. These underground hibernacula are at a fairly constant temperature of about 12–15°C throughout a 24-h cycle, which is substantially lower than the mean temperatures of the tree hollow-dwelling western *C. medius* at approximately 23°C [12]. We selected these dwarf lemurs because they are biologically close to western *C. medius* but mirror environmental conditions experienced by temperate hibernators (i.e. underground temperature-buffered hibernacula). Thus, these data will shed light on the possible role of environmental conditions in the expression of hibernation.

## 2. Material and methods

### 2.1. Study site and study animals

We conducted this study in a 200 ha forest fragment at Tsinjoarivo, one of the last remaining high-altitude rainforests in eastern Madagascar (19°41′15′′ S, 47°46′25′′ E, 1660 m) [13]. At this location, there are two sympatric dwarf lemur species, *C. crossleyi* (approx. 350 g) and *C. sibreei* (approx. 250 g). Crossley's dwarf lemurs have a broad geographical distribution and occupy a variety of eastern rainforests including degraded environments and littoral forests, whereas Sibree's dwarf lemurs are high-altitude specialists [29,30]. The duration of hibernation varies between species and across individuals, but it ranges between three and seven months a year. During the hibernation period (April–September), dwarf lemurs of both species retreat into the ground [13].

### 2.2. Procedures

#### 2.2.1. Trapping schedule and temperature data

Dwarf lemurs were captured in March 2012 and January–February 2013 using Tomahawk traps (Tomahawk Live Trap, Hazelhurst, WI, USA) baited with small pieces of fermented banana. Traps were set between 3–10 m high along known trails at 17.00 and checked the following morning at 4.00. All captured lemurs were brought back to the campsite, where they were identified/marked, weighed and measured. Captured individuals were released at the site of capture later the same day. A subset of captured individuals was collared to attach radio-transmitters with temperature sensors (Advanced Telemetry Systems, Isanti, MN, USA, ARC400, approx. 14 g) that recorded skin temperature (*T*_sk_) hourly. During hibernation, when individuals are curled up in a ball, transmitters are tightly attached to the neck area providing a reliable estimation of body temperature [31]. Data loggers (Maxim DS1922 iButton, Maxim Integrated Products, Inc., San Jose, CA, USA) were placed to record temperature of hibernacula (*T*_h_) as well as *T*_am_. Sleeping sites/hibernacula locations were recorded using telemetry (Receiver R410, Advance Telemetry System, Isanti, MN, USA) on a weekly basis between March and July 2012, and March and July 2013.

#### 2.2.2. Settings for hibernation studies

Radio-collared dwarf lemurs were retrieved from their natural hibernacula in July 2012 and 2013 and transferred to experimental boxes to conduct our study. These ‘boxes’ consisted either of a clear plastic container, approximately 30 × 20 × 10 cm, or a 20 cm length of PVC pipe of approximately 15 cm diameter, which could be sealed at each end. Both of these chambers were equipped with tubes at either end to allow for the air within to be pumped through an oxygen analyzer, as well as inflow of fresh air. Experimental boxes, with study animals connected to EEG electrodes (see below), were returned to hibernation sites and buried to approximate natural conditions of temperature and light levels. The animals were carefully monitored to ensure adequate airflow through the experimental chambers.

#### 2.2.3. Measurement of metabolic rate

Metabolic rate data were also obtained as in our prior work [26]. The rate of metabolism was measured as VO_2_ (rate of oxygen consumption) using a portable oxygen analyzer (OxBox, designed and constructed by T. Ruf & T. Paumann, FIWI, University of Veterinary Medicine Vienna) with chemo-electric sensors (Bieler & Lang, Achern, Germany; accuracy less than 0.02 vol%). Oxygen sensors were calibrated directly before the field season in the laboratory using calibration gas made by a gas-mixing pump (Wölsthoff, Bochum, Germany, type G27). The experimental boxes were connected with a gas-tight tube to the oxygen analyzer. Air flow was measured at 50 or 70 l h^−1^, depending on the size of the box. The flow rate was continuously monitored by the mass flow meter of the gas analyzer system. Sampled air was dried and filtered with silica gel before entering the gas analyzer. Oxygen content of the sample air was measured once per minute. To control for any drift of the oxygen sensor, reference air (surrounding air) was analysed once per hour for 5 min (zero check). Metabolic rate was calculated as millilitres O_2_ per hour. We also calculated mass-specific VO_2_ expressed as millilitres O_2_ per gram per hour. Torpor bouts could be identified by unambiguously depressed metabolic rate. Operationally, we define torpor when oxygen measurements were under 100 ml h^−1^ and arousals when oxygen measurements were above 100 ml h^−1^, in agreement with values reported previously [26].

#### 2.2.4. Electroencephalographic recording methods

EEG methods were the same as used in our prior work [28]. As an initial step, the animals were removed from their underground hibernacula for insertion of needle EEG electrodes. In one of the animals studied (‘Ly’), this was done solely with the aid of a topical anaesthetic, which was possible due to the persistently torpid state of the animal. Otherwise, to minimize distress, animals were anaesthetized using ketamine 10 mg kg^−1^ (Ketaject, 100 mg ml^−1^ Bioniche Teoranta, Inverin, Co. Galway, Ireland) and midazolam 0.25 mg kg^−1^ (midazolam 5 mg ml^−1^, Bedford Laboratories, Bedford, OH, USA). We recorded EEG data by placing four subdermal needle electrodes just under the skin of the scalp, and electrocardiogram (EKG) data (only in 2013 individuals) by placing a fifth subdermal needle in the interscapular area (Grass-Telefactor, West Warwick, RI, USA). Because animals have muscle overlying the cranium, electrodes inserted through the skin may have been embedded in the tissue beneath at times. Each electrode was inserted into each of the four quadrants of the cranium: right, left, rostral and caudal. In order to keep the leads in the original location, they were anchored with the use of an adhesive semipermeable membrane (Tegaderm, 3M Health Care, St. Paul, MN, USA). We then wrapped this membrane with flexible cohesive bandage (Webtear, Webster Veterinary) and bandage tape (Wet-Pruf® The Kendall Co., Mansfield, MA, USA). We left the leads in place and recorded data as long as we were able to record technically acceptable data. The EEG recording device used was a Philips-Respironics DGx with capacity to record four EEG/electromyographic (EMG) channels, two EMG channels and one EKG channel. The subdermal needle electrodes were connected directly to the recording devices. Compumedics hardware and software (Compumedics Inc., Victoria, Australia) were used to record and review the data and to convert the data into European data format (EDF). All EEG records were scored in 30 s epochs by ADK who is Board Certified in Sleep Medicine (American Board of Sleep Medicine, American Board of Psychiatry and Neurology) and EEG (American Board of Clinical Neurophysiology, American Board of Psychiatry and Neurology), as previously described [28]. During scoring procedures, ADK chose the best channels for sleep stage identification and removed all data segments which included movement artefacts. However, we note that unlike the standard scoring criteria, REM sleep was operationalized as 30 s epochs where there was at least one rapid lateral eye movement and there was evidence of diminished EMG activity [28,32]. Because of the absence of electro-oculographic data, lateral eye movements were identified on the basis of their unique topography (a reversal of phase is seen in EEG leads on the left and right sides of the head which share a common reference) and morphology [28]. The artefacted data then underwent spectral analysis using standard methods implemented with software written by ADK which has been validated against Matlab (MathWorks, Natick, MA, USA) software [33,34]. This included fast-Fourier transformation (FFT) carried out in 2 s epochs averaging over both time and frequency, yielding frequency power spectra in six bands: *δ*-(0.5–3.5 Hz), *θ*-(4.0–8.0 Hz), *α*-(8.5–12 Hz), *σ*-(12.5–16 Hz), *β*-(16.5–30 Hz) and *γ*-(30.5–60 Hz). For each band, we computed power in µV2 for each 30 s epoch. EEG Delta Power was graphed as a function of time during arousals to assess the evidence for sleep deprivation. *β* and *γ* EEG power was used as a proxy for muscle activity as an indicator of potential REM sleep.

### 2.3. Sample size

Nine dwarf lemurs (*C. crossleyi* four females; *C. sibreei* two females and three males) wearing radio-transmitters were retrieved from their underground hibernacula for this study. Of these nine, seven experienced a period of sustained torpor followed by a spontaneous arousal during the period of evaluation. Owing to the fact that EEG intelligibility varied across individuals, recordings from four dwarf lemurs (three *C. crossleyi* and one *C. sibreei*), deemed too fragmentary, were not included in our analysis. As a result, this report is focused on the data collected from five animals for which EEGs could be analysed during torpor bouts as well as during arousal periods (table 1). For each individual, a single arousal episode was measured. For individuals ‘Ra’, ‘Sy’ and ‘Am’ (*C. sibreei*), data from pre-arousal and post-arousal torpor bouts were available. For individuals ‘Ly’ (*C. crossleyi*) and ‘Ju’ (*C. sibreei*), data from pre-arousal torpor bouts were only available.

**Table 1.** Dwarf lemurs monitored during hibernation. Start and end dates indicate the period during which individuals were placed in artificial hibernacula.

species/ID	sex	body mass (g)	start date	end date
*C. crossleyi* ‘Ly’	female	400	8 July 2012	14 July 2012
*C. sibreei* ‘Ju’	male	286	10 July 2012	20 July 2012
*C. sibreei* ‘Am’	female	393	23 July 2013	1 Aug 2013
*C. sibreei* ‘Sy’	female	345	26 July 2013	1 Aug 2013
*C. sibreei* ‘Ra’	male	288	16 July 2013	26 July 2013

## 3. Results

**Figure 1.**
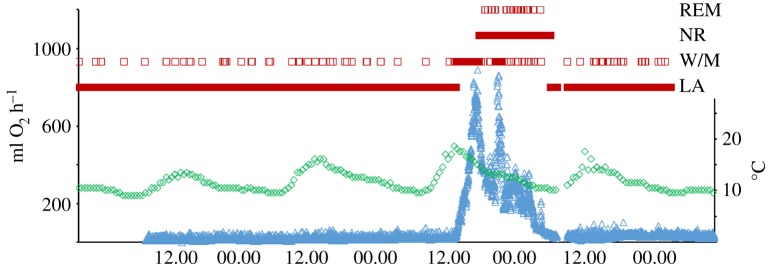
EEG profiles (squares) of individual ‘Am’ before, during and after an arousal, plotted against metabolic rates (triangles) and ambient temperature (diamonds). REM: rapid-eye movement stage, NR: non-REM stage, W/M: electric activity consistent with movement, low: low amplitude voltage.

**Figure 2.**
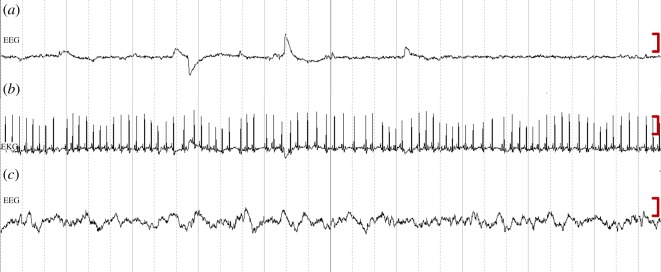
Three 30 s segments of EEG and EKG data. (*a*) EEG data during a period of REM sleep for individual ‘Am’; (*b*) EKG data during the same period for the same individual; (*c*) EEG data during a period of non-REM sleep for individual ‘Ra’. The display scale on the right is 75 µV.

**Figure 3.**
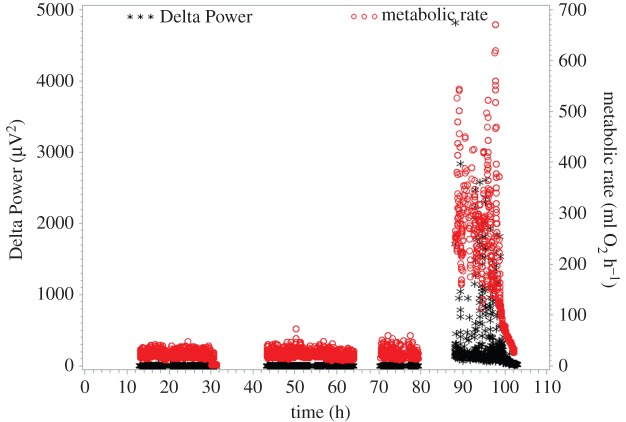
EEG Delta Power and metabolic rate in ‘Ju’, before and during arousal.

During torpor bouts, dwarf lemurs entered a state marked by a monotonous, ultra-low voltage EEG (figure 1). Physiological parameters during torpor bouts are shown in table 2. No EEG evidence of sleep in the usual sense was observed during torpor bouts at any time. During spontaneous arousals, individuals spent about half the time asleep (tables 2 and 3). These periods of sleep comprised both non-REM as well as REM sleep (figure 2). EEG recordings including the initiation of the arousal were available for four individuals (table 4) and following natural transition into torpor were available for three individuals (table 5). Non-REM was the first sleep stage occurring after the arousals began, but there was no evidence of a dramatic clustering that would potentially indicate substantial pent-up sleep need, such as may be expected following 3–5 days of total sleep deprivation. Figure 3 illustrates the continuous transition from relatively high Delta Power to the state of monotonous ultra-low voltage EEG, which occurs during torpor. During the entry into torpor, the EEG gradually transitioned from a pattern of non-REM sleep until achieving a sustained ultra-low voltage state. Because the transition is very gradual, the end of sleep and the beginning of torpor is rather arbitrary. It ranges from up to 5000 µV^2^ during the periods of arousal to remaining consistently under 10 µV^2^ throughout the periods of torpor. Metabolic rates during the first evidence of sleep is much higher than at the end, and non-REM sleep precedes REM at the onset of the arousal and follows REM-like states at the end (tables 4 and 5). Although first evidence of sleep occurred in all individuals at metabolic rates above 100 ml h^−1^, last evidence of sleep occurred at metabolic rates below 100 ml h^−1^. In all cases, sleep did not occur at *T*_sk_ below 25°C (tables 4 and 5).

**Table 2.** Percentages of sleep and ‘movement-wakefulness’ episodes during arousals. Arousals were defined by periods of metabolic rates above 100 ml O_2 _h^−1^.

ID	movement	non-REM	REM	duration (h)
‘Ly’	58	33	9	24^a^
‘Ju’	46	42	12	15^b^
‘Sy’	65	28	7	40.3
‘Am’	36	57	8	14.2
‘Ra’	57	38	6	5.5^c^

^a^EEG recordings missing for the last 7 h of the arousal.

^b^EEG recordings missing from first 4 h of arousal.

^c^EEG recordings missing for the last 4.5 h of the arousal.

**Table 3.** Mean and median (in parentheses) values for physiological parameters during torpor bouts. *T*_sk_, skin temperature; *T*_h_, hibernaculum temperature; HR, heart rate per minute; MR, metabolic rate; MSMR, mass-specific metabolic rate.

ID	*T*_sk_ (°C)	*T*_h_ (°C)	HR	MR (ml O_2 _h^−1^)	MSMR (ml O_2 _g^−1^ h^−1^)
‘Ly’	12.1 (12.1)	11.4 (11.7)	n.a.	14.8	0.04
‘Ju’	14.5 (14.5)	13.9 (13.6)	n.a.	21.3	0.07
‘Sy’	15.5 (14)	11.6 (11.7)	10 (8)	25.6	0.07
‘Am’	n.a.	11.6 (10.6)	12 (11)	21.2	0.05
‘Ra’	12.1 (12)	10.4 (9.7)	6 (6)	14.8	0.05

**Table 4.** Skin temperature (°C) and mass-specific metabolic rates (ml O_2_ g^−1^ h^−1^; in parentheses) during the first episode of wakefulness (W/M) and sleep (non-REM or REM) at the onset of an arousal.

ID	W/M	non-REM	REM
‘Ly’	13 (0.1)	25 (2.13)	30.5 (1.1)
‘Sy’	14.8 (0.17)	26.5 (1)	31.25 (0.84)
‘Am’	n.a. (0.10)	n.a. (1.46)	n.a. (0.74)
‘Ra’	12.3 (0.03)	30.3 (1)	32.5 (0.90)

**Table 5.** Skin temperature (°C) and mass-specific metabolic rates (ml O_2_ g^−1^ h^−1^; in parentheses), during the last episode of wakefulness (W/M) and sleep (non-REM or REM) at the end of an arousal.

ID	W/M	non-REM	REM
‘Ju’	32.5 (0.4)	25 (0.13)	27.5 (0.17)
‘Sy’	31 (0.64)	29.5 (0.22)	31 (0.54)
‘Am’	n.a. (0.40)	n.a. (0.11)	n.a. (0.15)

## 4. Discussion

This study provides the first analysis of EEG data during hibernation, including bouts of torpor and the subsequent spontaneous arousals in a primate hibernator. It also provides the first data on EEG during hibernation in high-altitude dwarf lemurs*.* The findings suggest that sleep does not occur during torpor bouts in these two lemur species, which are exposed to relatively cold temperatures, in contrast with the western species, *C. medius*. However, during arousals, non-REM and REM-like sleep both occurred in all animals studied.

### 4.1. Comparison with *Cheirogaleus medius*

Although we observed different findings from what we previously reported with *C. medius,* we believe that these differences are probably a reflection of the differing *T*_h_ in the animals studied rather than species differences. *Cheirogaleus medius* may hibernate in well-insulated tree hollows where temperature remains relatively constant around 23°C and spontaneous arousals have then been reported to occur after roughly 7 days of hibernation bouts [12]. However, we were only able to obtain EEG recordings from *C. medius* in poorly insulated hibernacula where the temperature fluctuates to a substantially greater degree and reaches much higher temperatures, leaving spontaneous arousals expendable. Unlike *C. crossleyi* and *C. sibreei* investigated in this study*,* the *C. medius* we studied did not maintain a sustained ultra-low voltage EEG during hibernation; instead, they manifested this monotonous ultra-low voltage pattern *only* at low ambient temperatures. However, when *T*_am_ (and consequently *T*_b_) rose to relatively high levels, this pattern would give way to a REM sleep-like state. On this basis, we hypothesize that there may be a link between sleeping and spontaneous arousals which may be driven by *T*_h_. If *T*_h_ is relatively high, at least several days in a week, spontaneous arousals are not needed and sleep occurs, whereas if *T*_h_ is continuously low, there is a need for the arousals and sleep does not occur during hibernation bouts.

### 4.2. Comparison with non-primate hibernators

The findings of our study suggest that many features of hibernation in *C. crossleyi* and *C. sibreei* mirror what is seen in non-primate hibernators such as the Arctic, golden-mantled, and European ground squirrels, including non-REM and REM sleep observed exclusively during thermogenically induced arousals, but not during hibernation bouts [17,20,21]. Studies in the ground squirrels also note a monotonous ultra-low voltage EEG occurring during hibernation bouts and that sleep does not occur unless the *T*_am_ is raised well above that normally experienced during hibernation [16,24]. In ground squirrels, higher *T*_am_ during hibernation was noted to be associated with non-REM sleep, but also with lessened EEG Delta Power, which has been postulated as evidence of sleep deprivation during arousals [16,17,35]. Unlike dwarf lemurs (at least the western *C. medius*), which can continue to hibernate at high *T*_am_, squirrels aroused when temperatures were artificially raised up to approximately 25°C [16]. As squirrels are not naturally exposed to these *T*_am_ during winter, the ability of lemurs to hibernate despite hot *T*_am_ may indicate inherent biological differences.

### 4.3. Future avenues

A missing link in the study of primate hibernation is the comparison of EEG in dwarf lemurs hibernating under stable but warm conditions with those hibernating in colder and variable environments. Recently, it was shown that tenrecs, the other endemic mammalian group known to hibernate in Madagascar, can do so underground for up to nine months without undergoing endogenously generated arousals [9]. Sandy soils, where tenrecs hibernated, never cooled down under 22°C or heated up above 30°C. Dwarf lemurs hibernating in well-insulated tree hollows (relatively stable *T*_h_ between 20 and 25°C) in western Madagascar do undergo arousals once a week, although the arousal bouts are shorter (approx. 6 h) than those of Arctic hibernators or high-altitude dwarf lemurs ([12]; this study). Thus, there may be species-specific temperature thresholds that trigger the energetically expensive arousals, which presumably allow critical metabolic processes to resume. These differences, if they exist, may be the result of unrelated evolutionary histories. Although tenrecs may have retained the ability to use torpor from their ancestors [9], cheirogaleids may have diverged from non-heterothermic lemurs and evolved hibernation more recently, after their diversification in Madagascar. If this is the case, investigating hibernation in lemurs, our closest relatives known to hibernate, may be particularly relevant to research concerned with induction of hibernation-like states in humans.

## Supplementary Material

Excel files (5) one per individual for which data were used C_crossleyi_Ly_database

## Supplementary Material

C_sibreei_Am_database

## Supplementary Material

C_sibreei_Ju_database

## Supplementary Material

C_sibreei_Ra_database

## Supplementary Material

C_sibreei_Sy_database

## Footnotes

1 Because definitions of torpor and hibernation vary in the literature, we use torpor bout and hibernation *sensu* Villanueva-Cañas *et al.* [3], i.e. torpor as controlled reduction in metabolic rates and body temperature and hibernation as the multiday expression of torpor including inter-bout arousals if present.

## Data accessibility

Our databases have been submitted as electronic supplementary material.
